# Synergistic Mechanisms Underlie the Peroxide and Coagent Improvement of Natural-Rubber-Toughened Poly(3-hydroxybutyrate-co-3-hydroxyvalerate) Mechanical Performance

**DOI:** 10.3390/polym11030565

**Published:** 2019-03-26

**Authors:** Xiaoying Zhao, Katrina Cornish, Yael Vodovotz

**Affiliations:** 1 The Ohio State University, Department of Food Science and Technology, 2015 Fyffe Road, Columbus, OH 43210, USA; zhao.1630@osu.edu; 2 The Ohio State University, Department of Horticulture and Crop Science, 1680 Madison Avenue, Wooster, OH 44691, USA; cornish.19@osu.edu; 3 The Ohio State University, Department of Food, Agricultural and Biological Engineering, 1680 Madison Avenue, Wooster, OH 44691, USA

**Keywords:** poly(3-hydroxybutyrate-co-3-hydroxyvalerate) (PHBV), bioplastic, natural rubber, reactive extrusion, toughening

## Abstract

Poly(3-hydroxybutyrate-co-3-hydroxyvalerate) (PHBV) is a promising bio-based and biodegradable thermoplastic with restricted industrial applications due to its brittleness and poor processability. Natural rubber (NR) has been used as a toughening agent, but further physical improvements are desired. In this study, rubber toughening efficiency was significantly improved through the synergistic use of a trifunctional acrylic coagent and an organic peroxide during reactive extrusion of PHBV and NR. The rheological, crystallization, thermal, morphological, and mechanical properties of PHBV/NR blends with 15% rubber loading were characterized. The peroxide and coagent synergistically crosslinked the rubber phase and grafted PHBV onto rubber backbones, leading to enhanced rubber modulus and cohesive strength as well as improved PHBV–rubber compatibility and blend homogeneity. Simultaneously, the peroxide–coagent treatment decreased PHBV crystallinity and crystal size and depressed peroxy-radical-caused PHBV degradation. The new PHBV/NR blends had a broader processing window, 75% better toughness (based on the notched impact strength data), and 100% better ductility (based on the tensile elongation data) than pristine PHBV. This new rubber-toughened PHBV material has balanced mechanical performance comparable to that of conventional thermoplastics and is suitable for a wide range of plastic applications.

## 1. Introduction

Currently, more than 90% of plastics are petroleum-based and non-biodegradable [1], and their wide industrial use is of significant environmental concern. Development of biodegradable polymers from renewable resources is needed to lessen oil dependence, reduce environmental pollution, and improve global sustainability. Polyhydroxyalkanoates (PHAs) are biodegradable aliphatic polyesters produced through bacterial fermentation of sugars or lipids [2]. PHA properties can be customized to meet the final use requirements by adjusting the monomer unit composition [3], microorganism, and carbon source used in fermentation [4]. PHA versatility provides a wide range of properties for various applications [5], contributing to the predicted market growth from about 51,150 tonnes in 2010 to 405,100 tonnes by 2020 [6].

One of the most studied PHAs is polyhydroxybutyrate (PHB), obtained from polymerization of 3-hydroxybutyrate monomers. It has properties similar to polypropylene (PP) but is stiffer and more brittle—characteristics which prevent its widespread industrial use. Incorporation of poly(3-hydroxyvalerate) to form poly(3-hydroxybutyrate-co-3-hydroxyvalerate) (PHBV), which has improved flexibility and toughness, has various promising applications in the packaging, biomedical, agricultural, automotive, and construction industries [7,8]. Yet, despite these enhancements, PHBV remains brittle, has an unacceptably narrow processing window, and is expensive [9]. PHBV brittleness is mainly caused by its high degree of crystallinity and large spherulites [10,11], which can be reduced by plasticization [12], incorporation of nucleating agents to reduce spherulite size [13,14], chemical modification, and blending with flexible polymers [15]. 

Incorporation of natural rubber (NR) into PHBV through melt blending is an industrially and economically viable route to improve PHBV flexibility and toughness. NR is a promising toughening agent due to its ductility, elasticity, availability, and low cost [16]. NR’s renewability and biodegradability can also help maintain the “green” nature of PHBV [16]. The rubber toughening mechanisms can be explained by several theories. According to void toughening theory, rubber particles concentrate stress then go through cavitation, releasing volume strain energy and reducing the resistance of the polymer matrix to volumetric expansion [17,18,19]. The matrix then undergoes shear yielding and/or crazing, which absorbs most of the fracture energy [17,18,19]. In contrast, stress field theory suggests that rubber particles influence the crystallization behavior of the polymer matrix and introduce a layer of oriented crystalline lamellae perpendicular to the rubber/matrix interface [20,21]. The lamellae are connected by hydrogen-bonded planes which are perpendicular to the lamellae and have low slip resistance [20]. When impacted by an outside force, the inter-particle regions deform through crystalline slip, causing shearing of the surrounding crystalline regions and rotation of the crystals, enabling more energy absorption and resulting in improved toughness [20]. This is also the concept behind filler-induced toughening of polymers [22]. 

In both theoretical models, NR’s toughening efficacy depends on interfacial adhesion between NR and PHBV, rubber particle size and dispersion, inter-particle distance (ligament thickness), rubber modulus, matrix properties, processing conditions, and other factors [17]. Generally, optimal particle size, homogeneous rubber dispersion, thin ligaments, and good rubber/matrix adhesion are required for maximum toughness. Reactive blending has been used for rubber-toughened thermoplastics by improving blend morphology and compatibility between rubber and plastic [23]. This process selectively cures rubber during its intimate melt mixing with a thermoplastic [24], preventing either phase from coalescing [25] and leading to excellent rubber dispersion in the plastic matrix [26]. In addition, crosslinking allows rubber to reach high strains under stress through the formation of strong fibrils [17]. Crosslinking also enhances interconnectivity among stress fields around rubber particles, allowing for easier stress transfer [17,27]. Covalent bonds between the rubber and plastic matrixes clearly would overcome interfacial repulsion and improve interfacial adhesion [28,29].

Peroxide-induced crosslinking of rubber and other polymers is an effective non-sulfurous crosslinking method acceptable in food contact applications [30]. Peroxides produce strong free radicals as they break down when exposed to heat during melt blending [30]. The radicals abstract H atoms from methylene groups on polymer chains and initiate formation of C–C crosslinks between very different, normally incompatible polymers [30]. Reactive blending of PHB and PBS (polybutylene succinate), with dicumyl peroxide (DCP) crosslinker, reduced PBS particle size and enhanced interfacial adhesion between the two polymers [31]. The PHB/PBS blends had improved tensile and impact strength over pristine PHB [31]. Similarly, PLA flexibility was improved by DCP-initiated reactive blending with NR [28]. Here, a continuous crosslinked NR phase was dispersed in the PLA matrix and the two phases had good interfacial adhesion (PLA grafted onto NR during melt-blending) [28]. However, peroxide-induced crosslinking lacks chemical selectivity, and competition between in situ compatibilization/crosslinking and free-radical-caused polymer degradation makes it difficult to control blend properties [32]. The balance between productive and non-productive competing reactions is affected by the polymer microstructure and the presence of dissolved oxygen and hydrogen donors (anti-oxidants, fatty acids, oils, etc.) in the formulation [32]. As most of the non-productive reactions are kinetically favored, productive reactions advancing effective crosslink formation can occur only if there is a very high concentration of reactive sites on the polymer backbone [32].

**Figure 1:**
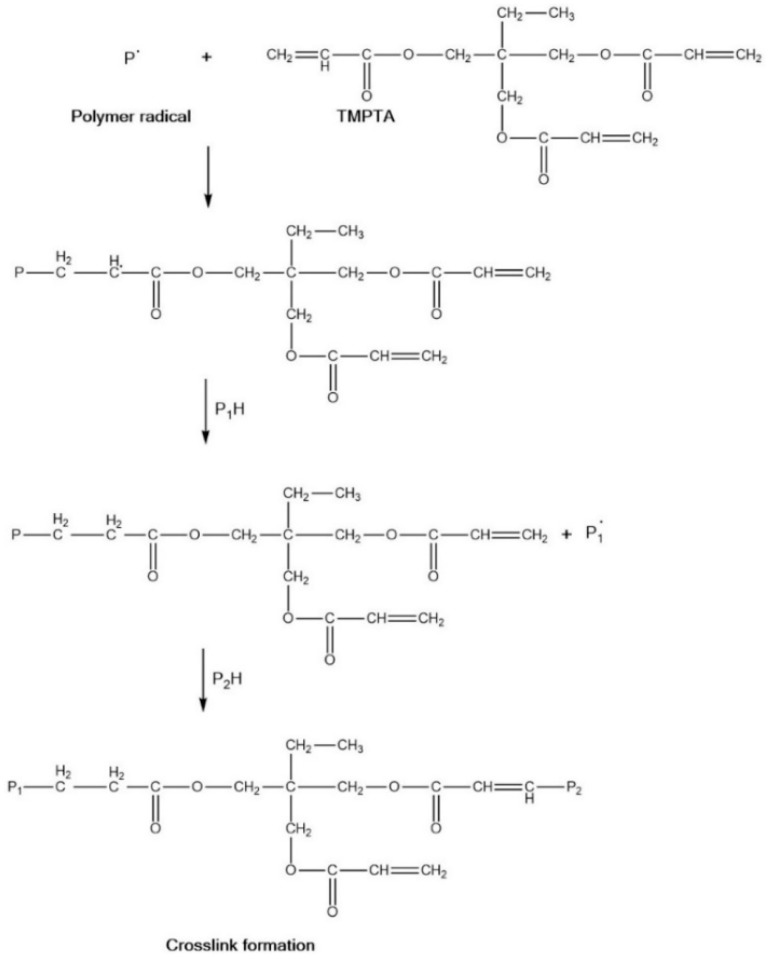
Trimethylol propane triacrylate (TMPTA) coagent reacts with polymer radicals [36,37].

Coagents are typically multifunctional monomers highly reactive with free radicals. Coagents can be used in peroxide-induced crosslinking to shift the reaction balance toward productive reactions by introducing a high concentration of reactive sites. These reactive sites result in efficient use of the peroxide-derived radicals and reduce the occurrence of deleterious side reactions [32], leading to increased crosslink density (Figure 1). The addition of coagents triallyl cyanurate (TAC), trimethylol propane triacrylate (TMPTA), and *N*,*N*′-m-phenylene dimaleimide (MPDM) in reactive blending of polypropylene (PP) and ethylene octene copolymer (EOC) increased the crosslink density of EOC and decreased the thermal degradation of PP [29]. The coagents limited deleterious PP chain scissions by reacting with PP macroradicals, forming a more stable polymer radical [29]. Also, the coagents reduced PP crosslinking by grafting onto PP polymer chains, which inhibited spherulite growth, decreased PP crystallinity, and improved PP toughness [29]. Similarly, the addition of coagent difunctional maleimide stabilized PP macroradicals and unsaturated polyester (UP) melts, localized the free radical reactions to PP/UP interfaces [33], maximized the formation of grafted copolymer, and improved blend properties [33]. 

In our previous study, peroxide alone was used during reactive blending of PHBV and NR to improve blend compatibility and morphology [34]. Blends containing 10–15% NR provided an optimal combination of processing and mechanical properties [35]. However, further improvements in blend strength and toughness are desired to obtain materials with balanced mechanical properties for broadened industrial applications. Therefore, a coagent was synergistically used with peroxide in this study to further improve the blend mechanical performance. The effect of the coagent on the thermal, crystallization, morphological, and mechanical properties of PHBV/NR blends was investigated. In our previous work [34,35], we investigated the effect of peroxide (0.15–0.45 wt %), coagent (0–0.63 wt %), and rubber (10–20 wt %) concentrations on the mechanical properties of PHBV/NR blends. The optimal formulation of the blends was 0.45 wt % peroxide, 0.63 wt % coagent, and 15 wt % NR, and this was used to fabricate blends of PHBV and NR in this work. 

## 2. Materials and Methods 

### 2.1. Materials

NR, which was Standard Indonesian Rubber-20 (SIR-20) of constant viscosity, was purchased from CentroTrade LLC (Wadsworth, OH, USA). Peroxide Luperox 101XL45 (2,5-Bis (tert-butylperoxy)-2,5-dimethylhexane) and coagent (trimethylolpropane triacrylate, TMPTA) were purchased from Sigma-Aldrich (St. Louis, MO, USA). PHBV with approximately 2 mol % hydroxyvalerate (HV) content was purchased from Tianan Biological Material Co. (Ningbo, China). Both rubber and peroxide were used as received. PHBV pellets were vacuum-dried for 24 h at 60 °C before use.

### 2.2. Methods

#### 2.2.1. Blend Preparation

Pre-dried PHBV, NR, peroxide Luperox 101XL45, and coagent TMPTA were premixed and fed into a pre-heated Leistritz ZSE-27 twin-screw extruder (Somerville, NJ, USA) for blending. The reactive blending was conducted at 60 rpm, with a reverse compounding temperature from 180 to 145 °C (Table 1). The formulae of the blends are shown in Table 2. The blends of PHBV and NR, with or without peroxide/coagent treatment, are generally referred to as PHBV/NR blends in this study.

#### 2.2.2. Gel Fraction Measurement

Pelleted samples were dissolved in chloroform at 60 °C for 24 h. The turbid solutions were centrifuged at 4000 rpm for 10 min. The gels on the top of the centrifuged solutions were collected, rinsed by chloroform five times, and dried. The gel fractions (*G*_f_) were calculated as follows: $G_{f}=m_{1}/m_{0}\times 100\%$, where $m_{0}$ is the initial weight of the samples and $m_{1}$ is the weight of the dried residue obtained after chloroform extraction [31].

#### 2.2.3. Rheological Characterization

The rheological behavior of the materials was analyzed using a TA Instrument Ares LSII rheometer (New Castle, DE, USA). Rheological determinations were performed at 175 °C using a 25 mm parallel plate system. Each disk sample, with a thickness of 1 mm and diameter of 25 mm, was equilibrated for 5 min before the gap was set to the testing position of approximately 0.9 mm or until the top plate made contact with the upper surface of the sample. The complex viscosity, storage modulus (*G*′), and loss modulus (*G*″) were measured with increasing frequency from 0.1 to 100 rad s^−1^ at 0.3% strain [38,39,40].

#### 2.2.4. Scanning Electron Microscopy (SEM)

The fracture surfaces of the samples from notched impact testing were visualized using a Quanta 200 (FEI Inc., Hillsboro, OR, USA) scanning electron microscope (SEM). The samples were observed after mechanical failure at ambient temperature. Samples were coated with a 10 nm layer of gold using a Cressington 108 sputter coater (Watford, UK).

#### 2.2.5. Mechanical Characterization

Pellets of PHBV/NR blends were injection molded into mechanical testing specimens and vacuum-dried for 24 h at 60 °C prior to testing. Tensile testing was conducted according to ASTM D638-08 using an Instron 5542 with the Bluehill v. 2.17 software package (Instron Corp., Norwood, MA, USA). Dumbbell-shaped samples (165.0 × 19.0 × 7.0 mm^3^) with a grip distance of 115 mm were prepared, and a crosshead speed of 5 mm·min^−1^ at room temperature was used. The flexural properties were determined according to ASTM D790-15. The test sample dimensions were 127.0 × 12.7 × 3.2 mm^3^. The 1% secant modulus, which represents the stress–strain ratio at the point on the curve that corresponds to the point of extension that is 1% of the initial sample length, was determined to evaluate the flexibility of the samples. Notched impact tests were conducted according to ASTM D256-10. Notched impact samples (63.5 × 12.7 × 0.32 mm^3^ with a 22.5° notch) were tested using an impact tester from Tinius Olsen (Horsham, PA, USA). The reported standard deviation (SD) values were calculated from at least three samples. Statistical analyses were performed using JMP 10.0 (Marlow, Buckinghamshire, UK). Significant differences (*p*-values < 0.05) in mechanical data between PHBV and its blends were determined using one-way analysis of variance (ANOVA) and the Tukey HSD method.

#### 2.2.6. Thermogravimetric Analysis (TGA)

A TA Instrument Discovery TGA 550 (New Castle, DE, USA) was used to study the thermal decomposition properties of the materials. Samples were heated under nitrogen from room temperature to 500 °C at 20 °C·min^−1^. Extrapolated onset (*T*_o_) and peak (*T*_p_) degradation temperatures were taken from the weight loss and derivative thermograms, respectively.

#### 2.2.7. Differential Scanning Calorimetry (DSC)

The thermal transitions of the materials were investigated using a previously described procedure [38,41] with a TA Instrument Discovery DSC 2500 (New Castle, DE, USA). The samples were first heated from room temperature to 200 °C at 10 °C·min^−1^, annealed at 200 °C for 4 min to remove the thermal history, subsequently cooled to −85 °C, held for 4 min, and reheated from −85 to 200 °C at 10 °C·min^−1^. The onset and peak crystallization temperatures (*T*_c(onset)_ and *T*_c(peak)_) were determined from the cooling scans. The glass transition temperature (*T*_g_), melting temperature (*T*_m_), enthalpy of fusion (Δ*H*_m_), and degree of crystallinity (*X*_c_) were determined from the second heating scans. *T*_m_ was determined at the peak value of the endotherms and *T*_g_ at the midpoint of the heat capacity changes (0–20 J g^−1^). Δ*H*_m_ was determined from the area under the endotherms using TRIOS Software v4.1.1.33073. The relative crystallinity of the blends was obtained by dividing Δ*H*_m_ by the enthalpy value of a theoretically 100% crystalline PHBV (146 J·g^−1^) taken from literature values [42,43,44,45].

#### 2.2.8. X-ray Diffraction (XRD)

XRD patterns of the samples were obtained using a Rigaku Miniflex 600 X-ray diffractometer (Woodlands, TX, USA) operating at 40 kV and 15 mA. Data were collected in the range of 2θ = 5°–60° at 2°·min^−1^. Crystal interplanar distances (d-spacing) (three families of lattice planes (020), (110), and (121) studied) were calculated using Bragg’s relationship [46,47]: λ=2d×sinθ, where d is the d-spacing, λ is the X-ray wavelength (1.5406 Å), and θ is the scattering angle. The crystal size was calculated by the equation $D=\frac{K\times \lambda }{\beta \times \cos\theta }$, where D is the crystal size, λ is the X-ray wavelength, β is the full width at half-maximum (FWHM) of the peak, θ is the scattering angle, and *K* is the Scherrer constant (0.9) [48].

#### 2.2.9. Fourier Transform Infrared Spectroscopy (FTIR)

FTIR absorption spectra of the crosslinked part (gel from the chloroform extraction) of the blends were obtained using an Agilent 4500a portable FTIR spectrometer (Santa Clara, CA, USA) in attenuated total reflectance (ATR) mode, with 4 cm^−1^ resolution, 64 scans, and spectral wavelengths ranging from 650 to 4000 cm^−1^. 

#### 2.2.10. Proton Nuclear Magnetic Resonance (^1^H NMR) Spectroscopy

The chemical structures of the uncrosslinked part (solution from chloroform extraction after removal of the gel) of the blends were characterized by ^1^H NMR (Bruker AC-P 400 MHz spectrometer) at room temperature with CDCl_3_ as a solvent. Chemical shifts were referenced to the residual proton peak of CDCl_3_ at 7.24 ppm.

## 3. Results and Discussion

### 3.1. Crosslink Formation during Reactive Blending

The crosslinking effect of the peroxide and coagent during the reactive blending of PHBV and NR is shown in Table 3. Of note, pristine PHBV, which did not go through the melt procedure, did not form a gel (crosslinks), as was expected, due to the lack of networks in its macro-molecular structure. Pristine NR had 1.5% gel, and extrusion did not break down the gel. The slightly higher gel content observed in extruded NR than in pristine NR may be caused by rubber aging under heat and oxygen [49]. Unexpectedly, no gel was present in PHBV/NR blend, indicating that the PHBV phase broke down the pre-existing NR gel and prevented the heat/oxygen-caused rubber aging/crosslinking during the melt blend formation. Both PHBV/NR/P and PHBV/NR/P/C contained gels mainly formed by the rubber phase, as indicated by the TGA thermograms and FTIR spectra of the gels (Figure 6; Figure 10, and discussed later). The PHBV/NR/P/C blend had a gel fraction similar to its rubber content, suggesting that the highly reactive coagent molecules fully crosslinked NR, potentially by restricting competing deleterious reactions through efficient use of peroxide-derived radicals. In contrast, the gel fraction of PHBV/NR/P (6%) was much lower than its rubber content (15%) but higher than the corresponding gel fraction of the incorporated rubber in the blend, indicating that peroxide alone, at 3 phr loading, only crosslinked a fraction of the rubber phase in this blend. It is worth mentioning that instead of synergistically using the coagent, increasing the loading of peroxide alone can also increase the NR crosslink density. However, the use of peroxide may be counterproductive because it can cause polymer chain scission, as discussed later.

### 3.2. Rheological Properties

**Figure 2:**
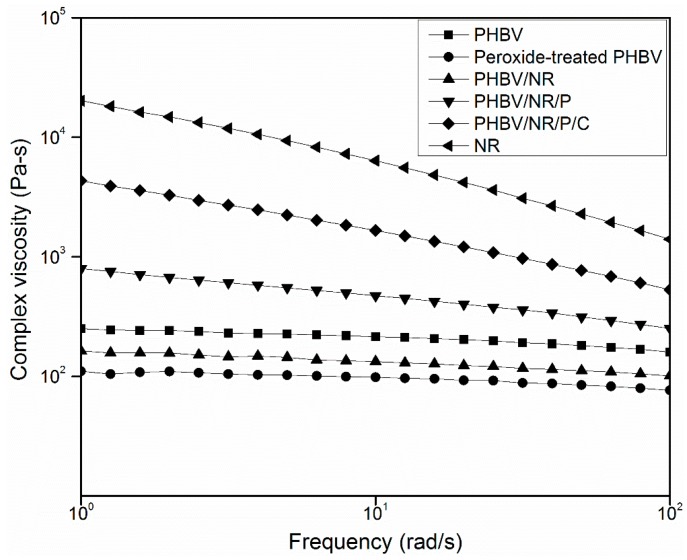
Complex viscosities of pristine PHBV, pristine NR, and PHBV/NR blends at 175 °C.

The formation of crosslinks during reactive blending significantly affected the rheological properties of PHBV in the blends. The viscosity of pristine PHBV decreased with peroxide treatment, mainly due to peroxide-caused polymer degradation through chain scission, which has been observed in peroxide-assisted reactive extrusion of polyolefin graft copolymers and peroxide-induced reactive blending of biodegradable polyesters poly-ε-caproIactone (PCL) and PHB [50,51]. PHBV/NR/P was more viscous and had more significant shear thinning behavior than PHBV/NR and pristine PHBV (Figure 2), suggesting (1) enhanced chain entanglements caused by peroxide-initiated crosslinking of NR in the blends [52], as supported by the higher gel content, and (2) covalent PHBV/NR grafting [53,54,55] (demonstrated by FTIR and NMR analysis and discussed below). This observation differs from a previous study where peroxide treatment decreased the viscosity of PP/ethylene–propylene rubber (EPR)/polyethylene (PE) blends by causing polymer chain scission [36]. The viscosity increase in PHBV/NR/P in this study indicated that peroxide crosslinking of NR more than compensated for any polymer chain scission [52]. PHBV/NR/P/C was more viscous than PHBV/NR/P (Figure 1), reflecting its greater crosslink density (gel content, Table 3) in the presence of coagent. The coagent played a triple role in this blend: (1) it promoted efficient NR crosslinking by covalently binding to NR polymer chains and forming a more stable NR polymer radical (Figure 1), which was more likely to form an NR–NR crosslink with another NR radical before deleterious scission or dehydrohalogenation reactions occurred [56]; (2) it crosslinked PHBV and NR to form copolymers (supported by FTIR and NMR results), reducing interfacial tensions and incompatibility between the two phases [36]; (3) during the melt blending process, peroxy radicals attacked the unstable tertiary H of PHBV molecules and caused chain cleavage, β chain scission, and chain branching [57,58], resulting in the formation of low-molecular-weight polymer chains [59,60,61] reflected by the decreased viscosity. It has been reported that a coagent can suppress this thermally enhanced degradation process by stabilizing and reacting with PHBV macroradicals (Figure 9) [52,57,62].

**Figure 3:**
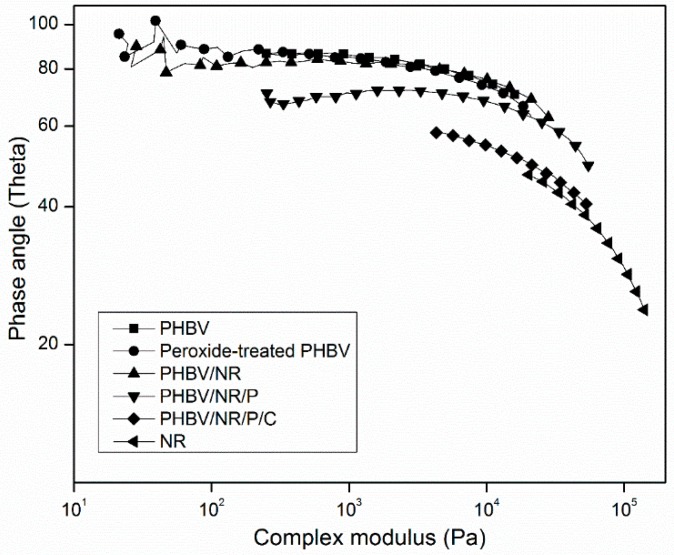
Van Gurp–Palmen plots of pristine PHBV, pristine NR, and PHBV/NR blends.

The crosslinking effect of peroxide and coagent was further studied by way of van Gurp–Palmen plots (Figure 3), which depict the relationship between the complex modulus (|*G*|*) and phase angle (δ) of materials. These plots detect the fluid–solid transition of polymer melts [63], with high δ values indicating dominant liquid-like behavior and low δ values indicating the formation of a more elastic system [63,64]. Pristine PHBV, peroxide-treated PHBV, and PHBV/NR had phase angles of approximate 90° at low |*G*|* values, reflecting their linear molecular chains with limited entanglements [65] and dominating fluid-like behavior. The observed high phase angle together with the previously discussed low complex viscosity of peroxide-treated PHBV indicated that a low peroxide content (3 phr) was insufficient to crosslink PHBV but was still able to degrade it. PHBV/NR/P had lower phase angles, indicating enhanced elastic behavior, likely caused by the formation of a partial rubber network reflected by its higher gel content than PHBV/NR. PHBV/NR/P/C had the lowest phase angle, similar to NR alone, indicating that coagent addition caused near-complete NR crosslinking, as demonstrated by its similar gel and rubber contents. The improved intraphase entanglements between NR and PHBV molecular chains were shown by FTIR and NMR analyses, discussed later. The rheological behavior of the PHBV/NR/P/C blend is consistent with its improved mechanical properties, which will be discussed below.

### 3.3. Mechanical Properties

**Figure 4:**
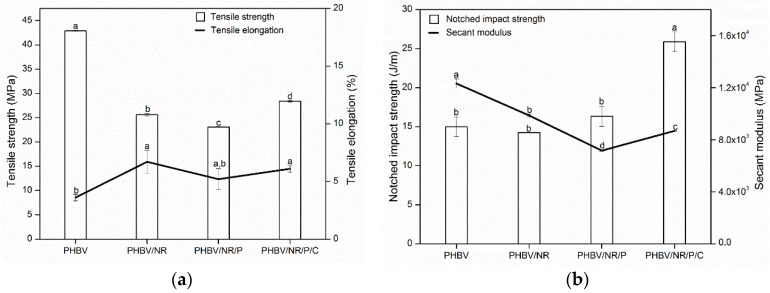
Mechanical properties of PHBV and the blends with NR. (Means showing different letters are significantly different at α = 0.05.)

Rubber addition decreased PHBV secant modulus, i.e., increased the flexibility, and increased tensile elongation (ductility) (Figure 4) by 40–100%. This is mainly due to NR’s liquid-like structural characteristic: the NR structure has no polar constituents. The van der Waals interactions between linear NR molecules cause coiled structures, leading to high flexibility and ductility [66,67]. The tensile strength of the blends was lower than that of pristine PHBV, mainly due to the low rubber modulus and strength (~0.3 MPa and 0.25 MPa, respectively [68]). Similar results were observed in most of the NR-toughened systems, such as PLA/NR and PP/NR blends [69,70]. Although the blend strength can be improved by enhancing interfacial adhesion between the two phases [18], rubber-addition-caused strength loss of the plastic matrix seems unavoidable. PHBV/NR/P/C was stronger than PHBV/NR and PHBV/NR/P, probably due to (1) coagent-induced rubber crosslinks and improved rubber modulus and strength, evinced by the high gel content and the high viscosity of PHBV/NR/P/C, respectively; and (2) increased intraphase entanglements between NR and PHBV, as discussed in the van Gurp–Palmen plots. Notched impact strength, i.e., toughness, was similar in PHBV, PHBV/NR, and PHBV/NR/P, but was 75% higher in PHBV/NR/C/P. This is probably again due to coagent-induced improvement in rubber crosslinks and interfacial bonding between PHBV and rubber, which helps absorb more energy and allow more stress transfer between the two phases during the fracture process [71,72]. In contrast, peroxide alone improved the notched impact strength of PLA/NR (60/40) and PP/NR (85/15) blends [53,70,73]. This difference may be attributed to PHBV having a significantly lower thermal stability than PLA and PP, and it undergoes substantial chain scission, which can be enhanced by the presence of peroxide, during melt blending, as proved by gel permeation chromatography (GPC), NMR, and mass spectrometry [59,60,61]. 

To summarize, the PHBV/NR/P/C blend had a combination of mechanical properties, i.e., tensile elongation of 6%, strength of 28 MPa, and notched impact strength (toughness) of 28 J·m^−1^, comparable to those of commercial thermoplastics such as general purpose polystyrene (tensile elongation of 3–9% [74,75], tensile strength of 25–34 MPa [76,77], and notched impact strength of 16–21 J·m^−1^ [78]), suggesting it could be used as a bio-substitute for conventional plastics. 

### 3.4. Morphology

**Figure 5:**
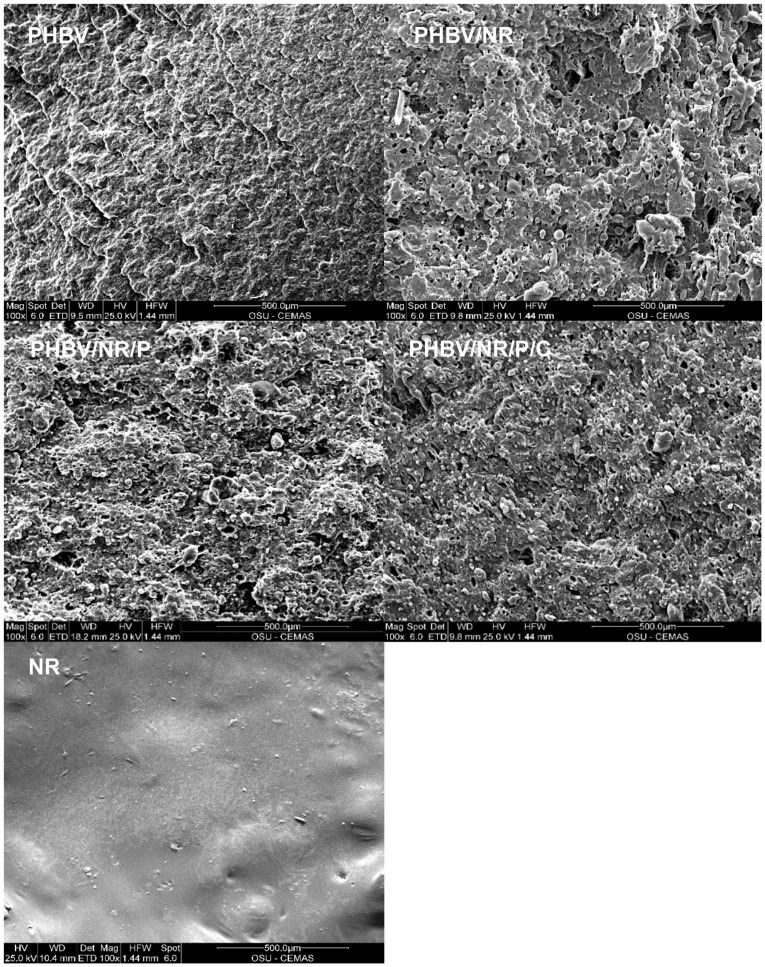
SEM images of the fracture surfaces of PHBV, NR, and the blends.

The morphology of the different samples (Figure 5) was as predicted from their mechanical behavior. PHBV had a fairly smooth surface, which is typical of brittle materials [79], yet NR also displayed a smooth surface although it is a ductile polymer [80,81,82]. The observed smooth surfaces can be ascribed to the different fracturing mechanisms of the two polymers, i.e., the innately ductile rubber mainly fails through yielding while the inherently brittle PHBV fails through crazing [83,84,85]. The three blends had much rougher surfaces than pristine PHBV, indicating improved ductility [86] as supported by their increased tensile elongation. Although it is not possible to distinguish NR and PHBV in the blends from the images, the lack of the “pulling out” phenomenon on the cross sections of the blends suggested adhesion between the two phases [86,87]. PHBV/NR/P and PHBV/NR/P/C had rougher fracture surfaces than PHBV/NR, corresponding to their higher ductility, supported by their increased tensile elongation, increased viscosity, and improved elastic behavior (Figure 2 and Figure 3). Such behavior is likely due to the peroxide and coagent enhancing interfacial adhesion between PHBV and NR. 

### 3.5. Thermal Properties

**Figure 6:**
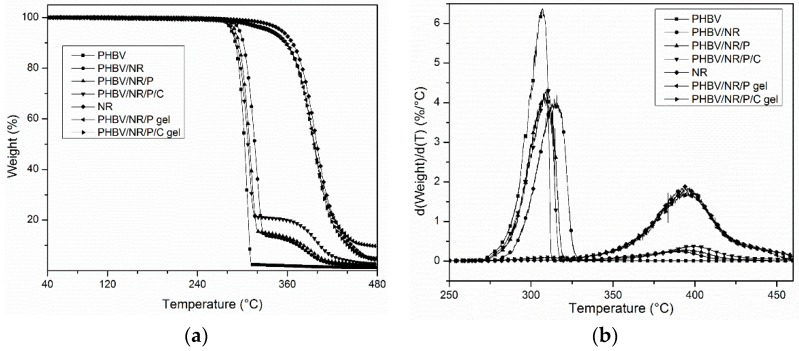
(**a**) Weight loss TGA thermograms; (**b**) Derivative weight loss TGA thermograms of pristine PHBV, pristine NR, blends of PHBV and NR, and the gels from PHBV/NR/P and PHBV/NR/P/C.

The three blends degraded in two stages (Figure 6), reflecting the different thermal stability of NR and PHBV. The first thermal degradation, between 285 and 310 °C, was associated with PHBV degradation, while the second stage, between 310 and 450 °C, was related to NR degradation [38]. The gels from PHBV/NR/P and PHBV/NR/P/C blends had TGA thermograms similar to that of NR, indicating that the gels from the two blends mainly contained rubber, while the slight decomposition around 300 °C may be associated with grafted PHBV. The similar onset thermal degradation temperatures (*T*_o_) of pristine PHBV and the PHBV phase in the blends indicate that some of the PHBV was not grafted to NR. However, grafted PHBV/NR was clearly observed due to its intermediate peak degradation temperatures (*T*_p_, Table 4). The presence of NR (both grafted and ungrafted) limited PHBV degradation during heating and rotational stresses of extrusion due to rubber dissipating heat more efficiently than PHBV. *T*_p_ − *T*_m_ of PHBV increased from 127 °C to 142, 138, and 139 °C in PHBV/NR, PHBV/NR/P, and PHBV/NR/P/C (Table 4), respectively, expanding the range of PHBV processing temperatures and broadening the processing window [9,88].

### 3.6. Crystallization Properties

#### 3.6.1. DSC

The three blends had two glass transition temperatures (*T*_g_) which corresponded to the *T*_g_ values of NR (−67 °C) and PHBV (5 °C), respectively (Table 4). The polymer melt temperature (*T*_m_) and degree of crystallinity (*X*_c_) decreased with increasing blend complexity. These changes, together with the slightly decreased crystallization temperatures of PHBV in the three blends, are consistent with the formation of imperfect crystallites, as observed previously in PHBV/nitrile blends [52,89]. These changes were likely due to two reasons: (1) the peroxide and coagent induced the formation of polymer networks, restricting PHBV crystallization, and (2) rubber particles migrating into the inter- and intra-spherulitic regions of crystalline PHBV, retarding crystal growth. Similar results were found in PP/EPDM/NR blends, where peroxide increased polymer chain crosslinks, particle size, and viscosity and interrupted the ability of PP molecules to align, further lowering the melt temperature and crystallinity [90]. All blends in this study were more flexible and less crystalline than pristine PHBV, but only PHBV/NR/P/C was tougher, even though both flexibility and toughness result from reduced crystallinity in brittle polymers [91,92].

#### 3.6.2. XRD

**Figure 7:**
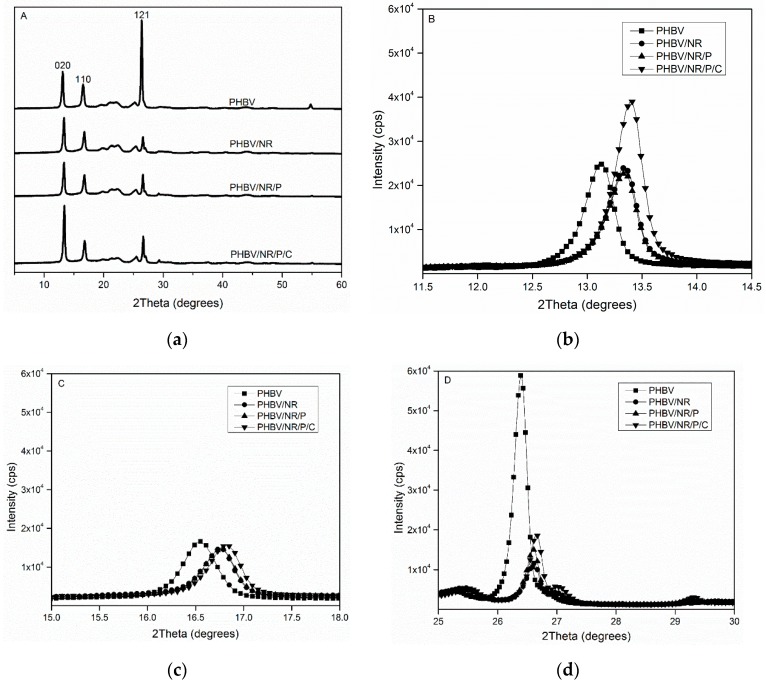
XRD patterns of pristine PHBV and the blends. (**a**) Overall XRD pattern from 2θ = 5° to 2θ = 60°; (**b**) XRD pattern of diffraction peak (020); (**c**) XRD pattern of diffraction peak (110); (**d**) XRD pattern of diffraction peak (121).

When the crystallization behavior of PHBV, alone and in the blends, was investigated by XRD, similar diffraction peaks [(020), (110), and (121)] indicating highly crystalline structures were observed (Figure 7a). However, the intensity of peaks (121) and, therefore, the degree of crystallinity dramatically decreased in the blends, confirming the DSC observations. No new peaks were observed in the XRD patterns of the blends, indicating that no new crystalline phases formed [93]. The (020), (110), and (121) peaks of PHBV shifted to higher angles in the blends, indicating decreased d-spacing (distance between atomic layers in a crystal), which was probably caused by lattice parameter changes [94], such as the formation of thinner crystals. PHBV/NR/P/C had the smallest d-spacing among the three blends, since increased crosslinks caused the greatest inhibition of crystal growth. PHBV crystal size, which was not related to the d-spacing but was inversely proportional to the FWHM of the peak [46,47,48], decreased from 25.5 to 23.5 nm in PHBV/NR/P/C while it was unchanged in the other blends. The small crystals may contribute to improved PHBV/NR/P/C toughness because they can reduce stress concentration. Highly concentrated stress is not desirable as it induces premature fracture, which increases the size of crystalline/amorphous phase interfaces and causes crack propagation [95,96]. Thus, small crystals improve toughness because they can enhance stress propagation and facilitate shear yielding of the plastic matrix [97,98].

### 3.7. Reaction Mechanism 

**Figure 8:**
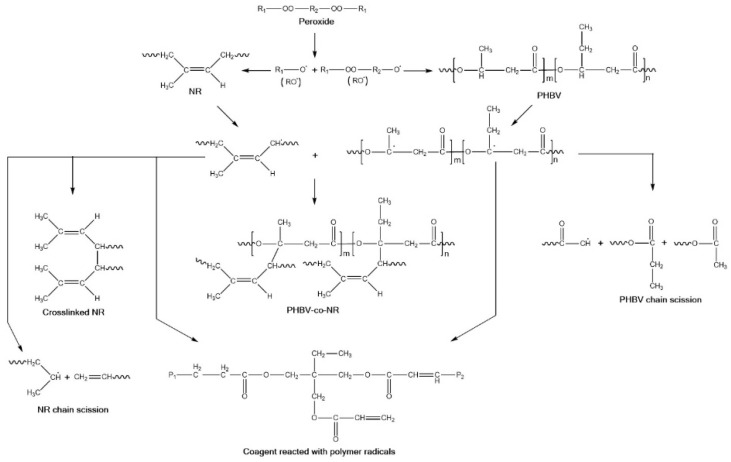
Possible reactions during melt blending of PHBV and NR in the presence of peroxide and coagent.

**Figure 9:**
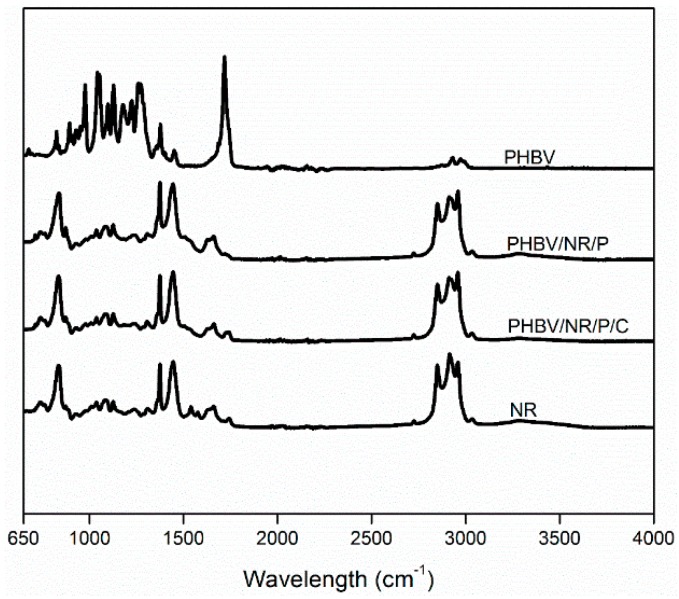
FTIR spectra of pristine PHBV, pristine NR, and gels from PHBV/NR/P and PHBV/NR/P/C blends obtained through chloroform extraction.

The gel and soluble fractions from chloroform extraction of the blends were characterized through FTIR and NMR, respectively, to investigate the reaction mechanism(s) of the coagent-assisted peroxide-induced reactive blending of PHBV and NR (Figure 8). The FTIR spectra of the gels were almost identical to those of pristine NR (Figure 9), indicating complete removal of free PHBV components during chloroform extraction of the blends.

#### 3.7.1. FTIR

Peaks at 1662 cm^−1^ (representing C=C bonds) were visible in NR and the gels from the blends (Figure 9). These may reflect unreacted NR double bonds and/or that rubber crosslinking occurring at the active α-H adjacent to the double bonds [99] (Figure 8). Peaks at 2957 cm^−1^ (representing C–H asymmetric stretch in CH_3_ groups) [100] were more intense in the gels from PHBV/NR/P and PHBV/NR/P/C than in NR, indicating grafting of PHBV and/or coagent onto NR backbones [101]. Peaks at 1539 cm^−1^ and 1576 cm^−1^, both representing N–H stretch, C=O, and N–H bending of amide groups of rubber proteins [102], were weakened in the blends, which may reflect protein degradation during the melt blending process. FTIR analysis indicated degradation of a particular fraction of the rubber proteins, for example, water-soluble and membrane-associated proteins [103]. The water-soluble proteins are the main immunogens in latex allergy [103].

#### 3.7.2. NMR

**Figure 10:**
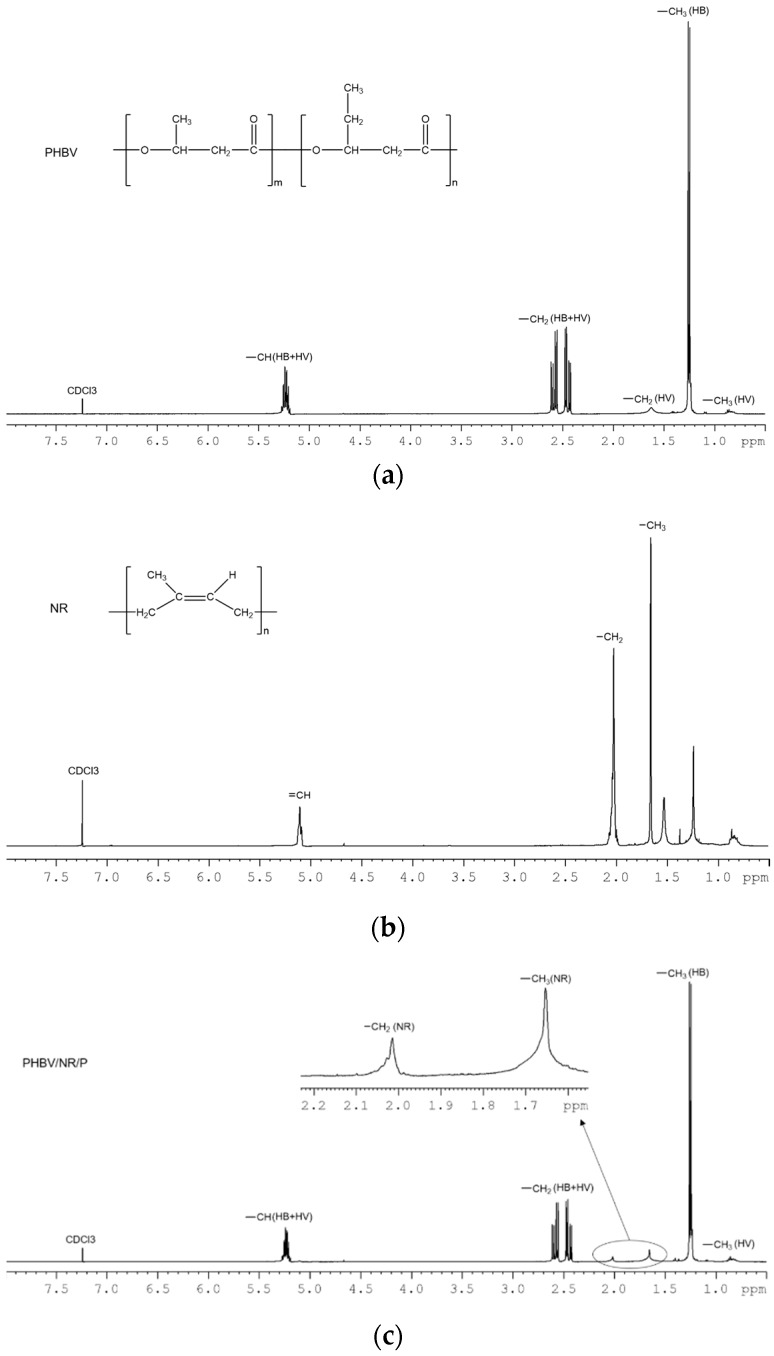

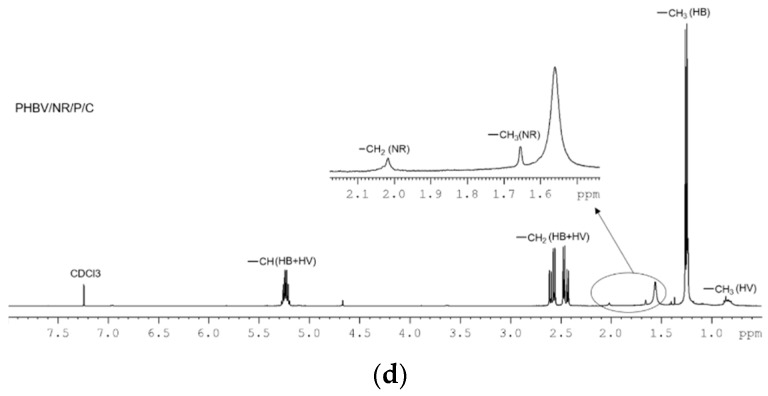
^1^H NMR spectra of (**a**) PHBV, (**b**) NR, (**c**) the chloroform-soluble part of PHBV/NR/P, and (**d**) the chloroform-soluble part of PHBV/NR/P/C.

^1^H NMR spectra of the chloroform-soluble fractions of PHBV/NR/P and PHBV/NR/P/C were similar to that of pristine PHBV (Figure 10), indicating that the soluble fractions mainly contained PHBV. Peaks at 2.02 and 1.65 ppm, belonging to the characteristic hydrogen protons of NR, were visible in PHBV/NR/P and PHBV/NR/P/C spectra, suggesting the formation of soluble PHBV-grafted-NR copolymers during the melt blending process. The peak at 1.56 ppm in the PHBV/NR/P/C sol spectrum may be caused by the water in the samples. No peaks belonging to the coagent were observed, indicating that most of the coagent units were in the blend gels. 

**Figure 11:**
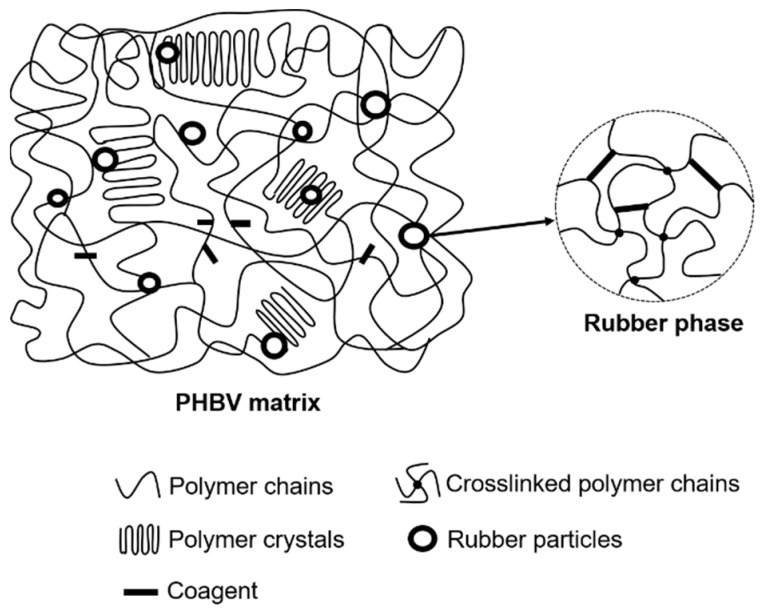
A conceptual structure of a coagent-assisted peroxide-cured PHBV/NR blend.

Thus, peroxide and coagent crosslinked the rubber phase in the blends. Grafting between PHBV and NR occurred during the melt blending process, forming two types of PHBV-grafted-NR structures (PHBV-co-NR, Figure 8): one locked within the rubber gel (indicated by the TGA analysis, Figure 5, and FTIR analysis, Figure 9) and one soluble in chloroform (indicated by the NMR analysis of the blend sols, Figure 10). Coagent bridges between two polymer chains formed and behaved similarly to reinforcing fillers in PHBV/NR blends (Figure 11) [37,65], improving matrix strength.

## 4. Conclusions

The rubber toughening efficiency of reactively extruded PHBV/NR blends was significantly improved through the synergistic use of coagent and peroxide, which crosslinked NR and grafted PHBV onto NR, resulting in increased interfacial adhesion, NR modulus, and cohesive strength while suppressing peroxide-induced thermal degradation of PHBV and decreasing PHBV crystallization. The new PHBV/NR blend had significantly improved toughness (by ~75%) and ductility (by ~100%) with minimal strength loss (~30%). This enhanced mechanical performance was much improved from that of other conventional dynamically vulcanized PHBV/NR blends, where rubber addition significantly decreased PHBV strength (by 40–80%) with only slight to moderate toughness improvement (by 10–50%) [35,38,104,105]. The new PHBV/NR material has mechanical properties (strength of 28 MPa and toughness of 28 J m^−1^) and processing windows comparable to those of some commercial plastics, such as PP and HDPE, and can replace some petroleum-based conventional thermoplastics in cast sheets and thermoforms, including those used in food packaging. Our insights into the reaction and toughening mechanisms of rubber-toughened PHBV lay the groundwork for future performance improvements.

## Figures and Tables

**Table 1:** Barrel temperatures of poly (3-hydroxybutyrate-co-3-hydroxyvalerate) (PHBV)/natural rubber (NR) reactive extrusion.

Heaters	Temperatures (°C)
1 (Below hopper)	180
2	175
3	175
4	170
5	170
6	160
7	160
8	155
9	150
10 (Die)	145

**Table 2:** PHBV and PHBV/NR blends obtained from extrusion melt blending.

Sample	PHBV/NR (wt/wt)	Peroxide (wt %)	Coagent (wt %)
PHBV	100:0	0	0
PHBV/NR	85:15	0	0
PHBV/NR/P	85:15	0.45	0
PHBV/NR/P/C	85:15	0.45	0.63

**Table 3:** Gel fractions of pristine PHBV, pristine NR, and PHBV/NR blends determined by chloroform extraction.

Samples	Pristine PHBV	Pristine NR	Extruded NR *	PHBV/NR	PHBV/NR/P	PHBV/NR/P/C
Gel (wt %)	0	1.56 ± 0.04	3.91 ± 0.02	0	6.0 ± 0.71	13.0 ± 0.35

* NR alone was extruded under the same conditions as the PHBV/NR blend extrusion.

**Table 4:** Melting, crystallization, glass transition, peak thermal degradation temperatures and degree of crystallinity of PHBV and NR and the blends determined from 2nd heating and 1st cooling of DSC at 10 °C min^−1^, respectively.

Sample	*T*_m_ (°C)	*T*_c(peak)_ (°C)	*T*_g(NR)_ (°C)	*T*_g(PHBV)_ (°C)	*T*_p_ (°C)	*X*_c_ (%)
PHBV	172.0 ± 0.04	120.8 ± 0.17	-	5.6 ± 0.30	299.4 ± 0.93	74.7 ± 0.02
PHBV/NR	171.9 ± 0.56	119.4 ± 0.59	−64.5 ± 0.52	6.0 ± 0.30	313.7 ± 1.49	61.6 ± 0.02
PHBV/NR/P	171.7 ± 0.26	117.2 ± 0.30	−65.2 ± 0.41	6.2 ± 0.30	309.5 ± 2.74	58.8 ± 0.02
PHBV/NR/P/C	169.8 ± 0.54	116.8 ± 0.33	−65.8 ± 0.54	4.3 ± 0.30	308.7 ± 1.63	56.8 ± 0.02
NR	-	-	−66.8 ± 0.08	-	394.7 ± 1.16	-

The column headings are *T*_m_, melting temperature; *T*_c(peak)_, peak crystallization temperature; *T*_g(NR)_, glass transition temperature corresponding to NR component; *T*_g(PHBV)_, glass transition temperature corresponding to PHBV component; *T*_p_, peak thermal degradation temperature; and *X*_c_, degree of crystallinity.
